# Delayed Hydrocephalus Following Listeria monocytogenes Meningitis in an Immunocompetent Child

**DOI:** 10.7759/cureus.104728

**Published:** 2026-03-05

**Authors:** Diana Guzmán García, Andrea A Sánchez Salgado, Jorge A Sánchez López, Jorge A García Campos, Carlos I Salazar Cerda

**Affiliations:** 1 Pediatrics, Hospital Regional, Instituto de Seguridad y Servicios Sociales de los Trabajadores del Estado (ISSSTE), Monterrey, MEX; 2 Pediatric Neurology, Hospital Regional, Instituto de Seguridad y Servicios Sociales de los Trabajadores del Estado (ISSSTE), Monterrey, MEX

**Keywords:** ependymitis, intracranial hypertension, listeria monocytogenes, obstructive hydrocephalus, pediatric meningitis, ventriculoperitoneal shunt

## Abstract

*Listeria monocytogenes* meningitis in immunocompetent pediatric patients may present as a severe central nervous system infection and requires prompt recognition and appropriate antimicrobial therapy. We report the case of a previously healthy twelve-year-old boy who presented with acute-onset fever, severe holocranial headache, nausea, and recurrent vomiting consistent with bacterial meningitis. Given his stable neurological examination without focal deficits or signs of increased intracranial pressure, brain magnetic resonance imaging was performed as the initial neuroimaging study and demonstrated diffuse leptomeningeal enhancement without ventricular dilation, with radiologic findings compatible with ependymitis. Empiric intravenous ceftriaxone and vancomycin were initiated. Cerebrospinal fluid culture subsequently confirmed *Listeria monocytogenes*, and antimicrobial therapy was adjusted to targeted intravenous ampicillin, resulting in defervescence within 48 hours, complete resolution of headache and vomiting, and a progressive decrease in inflammatory markers.

One week after hospital discharge, the patient returned with worsening headache, persistent vomiting, and anisocoria, consistent with acute increased intracranial pressure. A non-contrast head computed tomography scan performed at a secondary-level medical unit revealed obstructive hydrocephalus. Given the presence of neurological deterioration and radiological findings, repeat imaging was not performed, and the patient was transferred directly to the operating room for urgent ventriculoperitoneal shunt placement. The postoperative course was favorable, with resolution of anisocoria and progressive improvement in headache and vomiting. Neurological stability was maintained during follow-up. This case highlights the potential for delayed hydrocephalus following apparent clinical recovery from Listeria meningitis and underscores the importance of microbiological confirmation, timely antimicrobial adjustment, and structured neurological follow-up.

## Introduction

*Listeria monocytogenes *is a foodborne pathogen responsible for invasive infections, including meningitis and septicemia. Although the infection predominantly affects neonates, elderly individuals, and immunocompromised patients, sporadic cases have been described in immunocompetent children [1,2]. Reports of invasive disease in otherwise healthy pediatric patients continue to emerge [3]. While the overall incidence remains low, the clinical impact of Listeria meningitis in children may be disproportionate to its rarity [4].

Diagnostic challenges arise from atypical cerebrospinal fluid findings and from the organism's intrinsic resistance to third-generation cephalosporins, which constitute the first-line empiric therapy for suspected bacterial meningitis in children [5]. Because *Listeria monocytogenes* is not reliably covered by these agents, delayed recognition may lead to persistent central nervous system inflammation and severe complications.

Hydrocephalus represents an uncommon but serious complication of bacterial meningitis and may develop even after apparent clinical improvement. Persistent inflammatory injury affecting cerebrospinal fluid circulation has been proposed as the underlying mechanism [6]. We present the case of a previously healthy child with *Listeria monocytogenes* meningitis complicated by delayed hydrocephalus following apparent clinical recovery.

## Case presentation

A previously healthy twelve-year-old boy with a history of controlled intermittent asthma presented with a 24-hour history of sudden-onset severe holocranial headache (pain score 9/10), described as sharp and exacerbated by movement, associated with non-quantified fever, nausea, and recurrent vomiting unresponsive to oral analgesics and antipyretics.

He initially sought medical attention at a primary care facility, where marked leukocytosis and clinical signs suggestive of meningeal irritation were documented. Given the severity of symptoms, suspected central nervous system infection, and the need for advanced neuroimaging and specialized management, he was referred to a tertiary referral center for further evaluation.

Upon admission, the patient was somnolent and irritable, with a persistent severe headache and nuchal rigidity. He was hemodynamically stable with a temperature of 37.1° C (under antipyretic treatment), blood pressure of 137/80 mmHg, heart rate of 118 beats per minute, and oxygen saturation of 98% on room air. His Glasgow Coma Scale score was 15, and no focal neurological deficits were observed. Initial laboratory evaluation confirmed marked leukocytosis with neutrophilia and elevated inflammatory markers (Table 1).

**Table 1:** Relevant laboratory findings at admission Reference ranges correspond to age-adjusted pediatric laboratory standards for a 12-year-old patient. WBC - white blood cells; ESR - erythrocyte sedimentation rate; CRP - C-reactive protein

Test	Result	Reference range (12-year-old)	Units
Leukocytes (WBC)	39.55	4.5-13.5	×10³/µL
Neutrophils	37.1	1.8-8.0	×10³/µL
Lymphocytes	0.99	1.2-5.2	×10³/µL
Hemoglobin	13.3	12-16	g/dL
Hematocrit	39.9	36-46	%
Platelets	442	150-450	×10³/µL
ESR (VSG)	20	<10	mm/h
CRP	72.6	<5	mg/L

**Figure 1:**
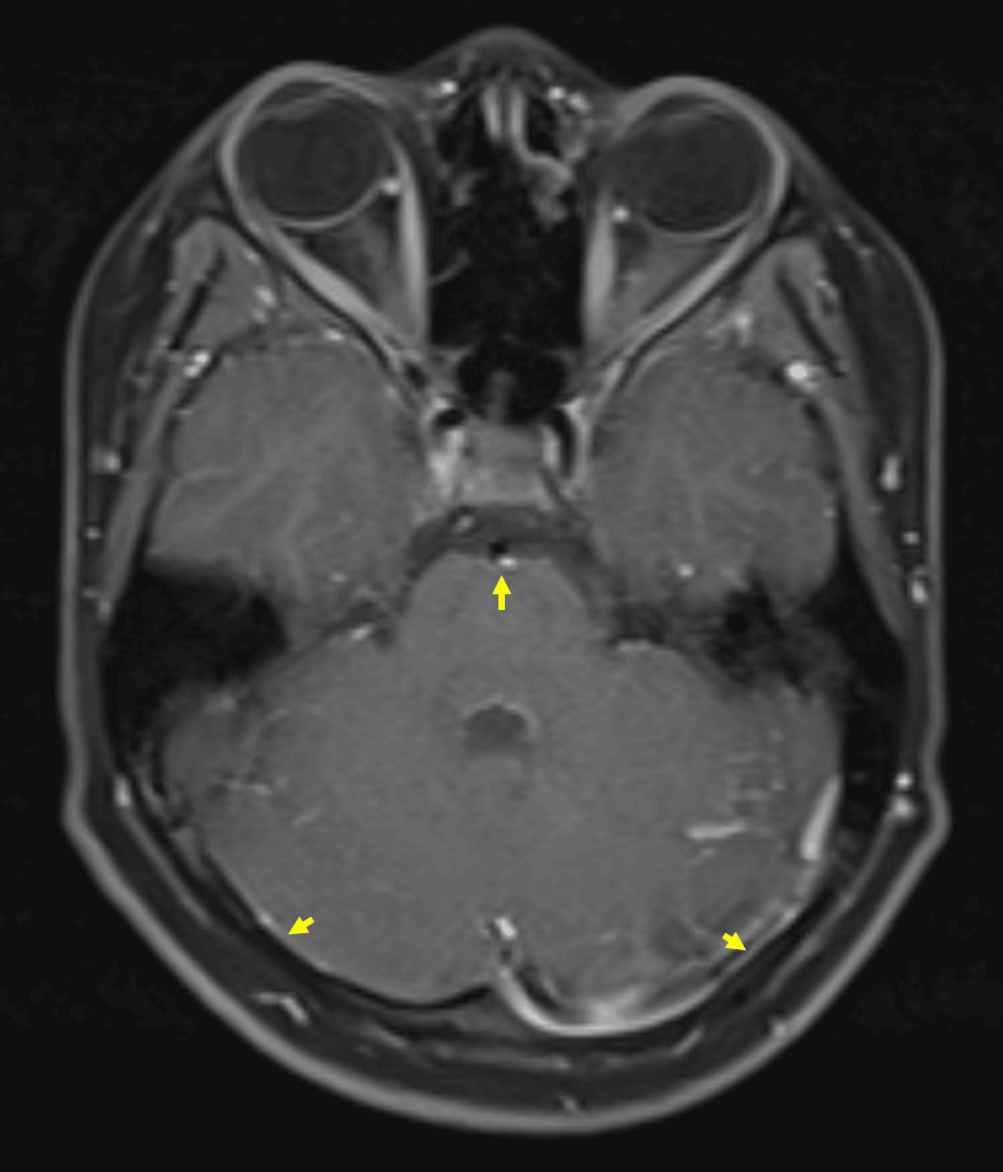
Axial T1-weighted post-contrast brain MRI Diffuse basal leptomeningeal enhancement (arrows), predominantly involving the prepontine and posterior fossa cisterns, consistent with acute bacterial meningitis.

Given his stable neurological examination without focal deficits, brain magnetic resonance imaging (MRI) was performed as the initial neuroimaging study. The MRI demonstrated diffuse leptomeningeal enhancement with hyperintense signal changes along the ependymal lining of the fourth ventricle, findings suggestive of meningitis with associated ependymitis (Figure 1).

Lumbar puncture was subsequently performed without complication. Cerebrospinal fluid analysis revealed neutrophilic pleocytosis, elevated protein concentration, reduced glucose levels, and increased lactate levels consistent with acute bacterial meningitis (Table 2).

**Table 2:** Cerebrospinal fluid analysis Reference ranges represent standard pediatric cerebrospinal fluid values. CSF - cerebrospinal fluid

Parameter	Result	Reference range	Units
Appearance	Slightly turbid	Clear	-
Leukocytes	1050	0-5	cells/µL
Neutrophils	85%	<5%	%
Lymphocytes	15%	40-80%	%
Red blood cells	0	0	cells/µL
Protein	145	15-45	mg/dL
Glucose	45	50-80	mg/dL
Lactate	5.8	1.1-2.4	mmol/L

Empiric intravenous ceftriaxone (80 mg/kg/day IV divided every 12 hours) and vancomycin (60 mg/kg/day IV divided every six hours) were initiated on the first hospital day to provide broad-spectrum coverage for common bacterial pathogens, including resistant organisms, in accordance with standard management guidelines for acute bacterial meningitis. Adjunctive dexamethasone (0.15 mg/kg per dose IV every six hours) was initiated concurrently with antibiotic therapy at presentation and administered for a total duration of five days as part of standard empiric management.

On the third hospital day, cerebrospinal fluid culture confirmed *Listeria monocytogenes*. Because this pathogen is more frequently associated with immunocompromised hosts, a comprehensive evaluation was performed to exclude underlying conditions. The patient underwent oncologic screening and immunologic assessment, including quantitative immunoglobulin levels and lymphocyte subset analysis, all of which were within normal age-adjusted reference ranges. No evidence of primary or secondary immunodeficiency was identified.

Due to the organism's intrinsic resistance to cephalosporins and in accordance with local epidemiological considerations, antimicrobial therapy was promptly adjusted to targeted intravenous ampicillin (200 mg/kg/day IV divided every six hours). The patient demonstrated progressive neurological improvement and completed a 21-day course of directed antibiotic therapy. He was discharged on hospital day 21 in stable condition without focal neurological deficits.

**Figure 2:**
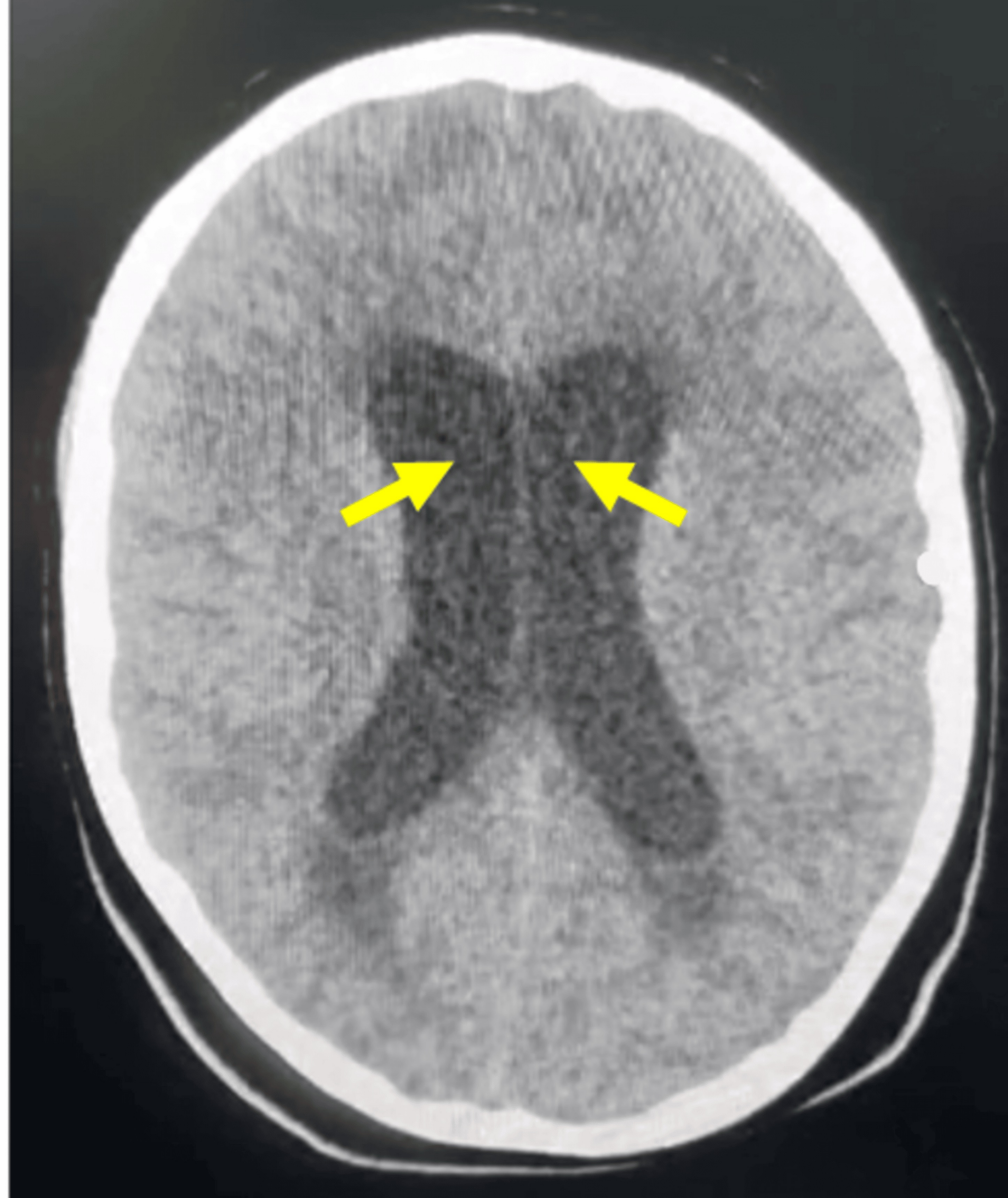
Axial head CT demonstrating obstructive hydrocephalus Axial computed tomography showing marked dilation of the lateral ventricles, particularly involving the frontal horns (arrows), consistent with obstructive hydrocephalus during the second hospitalization.

Seven days after discharge (day 28 from initial symptom onset), the patient returned with recurrence of severe headache and projectile vomiting. A non-contrast head computed tomography (CT) scan performed at a secondary-level hospital prior to transfer demonstrated ventricular dilation consistent with obstructive hydrocephalus (Figure 2).

He rapidly developed signs of acute intracranial hypertension, including anisocoria and Cushing response. Emergent ventriculoperitoneal shunt placement was performed. Due to the urgency of the clinical scenario, a repeat MRI was not obtained prior to surgical intervention.

**Figure 3:**
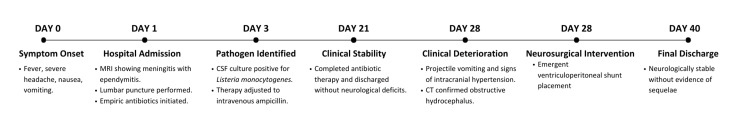
Clinical timeline of presentation, treatment, and complications Chronological summary of symptom onset, diagnostic evaluation, antimicrobial adjustments, development of delayed hydrocephalus, and neurosurgical intervention. CSF - cerebrospinal fluid

The postoperative course was favorable. The patient remained neurologically stable and was discharged on day 40 from initial presentation without evidence of neurological sequelae during follow-up (Figure 3).

## Discussion

*Listeria monocytogenes* is an uncommon but clinically significant cause of bacterial meningitis in pediatric patients. Although it predominantly affects neonates, elderly individuals, and immunocompromised hosts, invasive infection may also occur in previously healthy children without identifiable predisposing conditions [1,3]. Dietary exposure is frequently implicated; however, in many pediatric cases, no clear source is identified [1]. Because listerial infection is more commonly associated with immunodeficiency, clinicians should actively evaluate for underlying risk factors, including malignancy, immunosuppressive therapy, primary immunodeficiency disorders, and chronic systemic disease. In our patient, a comprehensive oncologic and immunologic evaluation was normal, supporting the diagnosis of invasive listeriosis in an immunocompetent host.

The intrinsic resistance of *Listeria monocytogenes *to third-generation cephalosporins complicates empiric management, as these agents remain standard first-line therapy for suspected bacterial meningitis [5]. For this reason, delayed recognition may contribute to persistent central nervous system infection and increased risk of complications [6,7]. Empiric addition of vancomycin is commonly recommended in severe presentations to ensure adequate coverage for resistant pathogens while awaiting microbiological confirmation [7,8]. Once Listeria is identified, ampicillin remains the cornerstone of targeted therapy and demonstrates favorable global susceptibility patterns [9]. Alternative therapeutic options described in the literature include trimethoprim-sulfamethoxazole or meropenem in cases of beta-lactam allergy or intolerance. Early adjustment to pathogen-directed therapy has been associated with improved clinical outcomes [9,10].

Large cohort data further confirm the significant morbidity associated with invasive listeriosis, particularly when diagnosis or antimicrobial adjustment is delayed [11].

Hydrocephalus represents an uncommon yet serious complication of bacterial meningitis and has been reported in association with Listeria infection in both immunocompromised and immunocompetent pediatric patients. In previously published pediatric cases, neurosurgical intervention was required due to progressive ventricular dilation despite appropriate antimicrobial therapy [2,3]. The most widely accepted mechanism involves persistent inflammatory injury affecting the ventricular system and subarachnoid pathways, leading to obstruction of cerebrospinal fluid circulation [12]. Residual inflammatory debris and fibrosis may impair cerebrospinal fluid outflow despite apparent microbiological clearance, explaining the development of delayed hydrocephalus even after initial clinical improvement.

Importantly, delayed hydrocephalus may occur after apparent clinical stability at discharge. This underscores the need for structured neurological follow-up, early reassessment in the presence of recurrent symptoms, and a low threshold for repeat neuroimaging in patients recovering from listerial meningitis.

## Conclusions

*Listeria monocytogenes *should be considered in pediatric meningitis cases that fail to respond to cephalosporin-based empiric therapy, even in immunocompetent patients. Delayed recognition may result in progression of central nervous system inflammation and the development of severe complications. Early microbiological evaluation, including cerebrospinal fluid and blood cultures, is essential to ensure prompt pathogen identification and timely initiation of targeted antimicrobial therapy, thereby reducing neurological morbidity.

Delayed hydrocephalus may occur after apparent clinical recovery and warrants vigilant neurological follow-up. Multidisciplinary coordination among pediatrics, infectious disease specialists, and neurosurgery is important for optimizing clinical management and minimizing the risk of long-term sequelae.
